# Trends in the prevalence of obesity among young Thai men and associated factors: from 2009 to 2016

**DOI:** 10.1186/s40779-019-0201-1

**Published:** 2019-04-30

**Authors:** Panadda Hatthachote, Ram Rangsin, Mathirut Mungthin, Boonsub Sakboonyarat

**Affiliations:** 1 Department of Physiology, Phramongkutklao College of Medicine, Bangkok, 10400 Thailand; 2 Department of Military and Community Medicine, Phramongkutklao College of Medicine, Bangkok, 10400 Thailand; 3 Department of Pharmacology, Phramongkutklao College of Medicine, Bangkok, 10400 Thailand

**Keywords:** Trends, Prevalence, Obesity, Young men, Thailand, The Royal Thai Army

## Abstract

**Background:**

The prevalence of obesity has been increasing in both males and females worldwide. In Thailand, the National Health Examination Surveys reported that the prevalence of obesity (body mass index (BMI) ≥30 kg/m^2^) among Thai male adults aged 20–59 years increased from 1.7% in 1991 to 6.8% in 2009. Obesity has been confirmed to lead to health problems, including noncommunicable diseases. In the present study, we report trends in the prevalence of obesity among new conscripts from 2009 to 2016. We also investigated the associated factors of obesity.

**Methods:**

Serial cross-sectional studies were conducted from 2009 to 2016 among male Royal Thai Army (RTA) conscripts whose weight and height had been measured to determine BMI after being inducted. Each subject completed a detailed risk factor questionnaire. Obesity was defined as BMI ≥ 30 kg/m^2^.

**Results:**

A total of 26,540 young Thai males conscripted into the RTA were included in this study. The prevalence of obesity was 2.2% in 2009, 3.4% in 2010, 2.5% in 2011, 2.9% in 2012, 3.4% in 2013, 4.4% in 2014, 5.0% in 2015, and 4.8% in 2016 (*P* for trend < 0.0001). The independent risk factors for obesity were coming from the north central and south regions compared with the northeast, higher education level, indoor occupation and no regular exercise.

**Conclusions:**

Our data emphasized that obesity constitutes a serious problem among young Thai men. We could apply these findings in military units to other groups at any age. Regular exercise should be provided to young adults and other age groups to slow the process of obesity, so that associated complications, especially noncommunicable diseases, will cease.

## Background

The prevalence of obesity has been increasing both in males and females worldwide [1, 2]. Globally, in 2013, the estimated prevalence of adults with a body mass index (BMI) ≥30 kg/m^2^ was 10% among males and 13% among females. The estimated prevalence of obesity (BMI ≥ 30 kg/m^2)^ among men aged ≥20 years in 2013 was 31.7, 27.5, 24.5, 13.2, 3.8 and 4.8% in the US, Australia, UK, Central African Republic, East Asia and Southeast Asia, respectively [2]. In Thailand, the National Health Examination Surveys reported that the prevalence of obesity (BMI ≥ 30 kg/m^2^) among Thai male adults aged 20 to 59 years increased from 1.7% in 1991 to 6.8% in 2009 [3]. Obesity is confirmed to lead to health problems, including hypertension [4, 5], diabetes mellitus and especially atherosclerotic cardiovascular diseases (ASCVD) [6, 7]. ASCVD may present as coronary artery disease, cerebrovascular accident and peripheral artery disease. In most cases, ASCVD results from obesity, primarily from complications of diabetes mellitus. Moreover, obesity can lead to ASCVD from multifactorial components associated with metabolic syndrome [8, 9]. The results of the Southern Community Cohort Study in the US indicated that young adults with obesity had an increased risk for all-cause mortality with hazard ratio (HR) = 1.64 and an increased risk of mortality from cardiovascular disease with a HR = 1.87 compared with the risks observed in normal weight adults[10]. Thus, when the process of obesity is interrupted, these complications will cease. In the present study, we reported trends in the prevalence of obesity among new conscripts from 2009 to 2016. We also investigated the pattern of associations between obesity and educational level, occupation, geographic area and behavioral factors from 2009 to 2016. We expected that the results of the study will represent the true situation of obesity among young Thai males.

## Methods

### Study design

The study comprised a serial cross-sectional survey and was conducted from 2009 to 2016. The data were retrieved from the database: a Surveillance in Heat Related Illness in Basic Military Training among new conscripts of the Royal Thai Army (HRI-RTA) after obtaining permission from the Military Medicine Research Unit of Phramongkutklao College of Medicine. The HRI-RTA evaluation surveillance was a cross-sectional survey aiming for the daily screening and surveillance of signs of heat accumulation and signs of heat-related illness among new conscripts during basic military training for 10 weeks [11]. The HRI-RTA was conducted in 35 military units over the 4 geographic regions of Thailand: (1) Bangkok Metropolitan Area and Lopburi Province in central Thailand; (2) Ubon Ratchathani Province in the northeast; (3) Chiang Mai Province in the north; and (4) Songkhla Province in the south.

### Subjects

Young Thai males aged 21 years were selected by the RTA for conscription using a lottery system. The lottery system was held in April annually at the district level of each province*.* Approximately 1 in 10 young males participate in the conscription annually where they register and participate in the conscription in the district in which their family resides. Moreover, males who were certain religious personnel, physically challenged, severely ill, or who were participating in alternative military service and some teachers were exempt.

The total number of participants included approximately 90,000 new conscripts annually. Induction occurs either in May or November of each year for the two-year duration of military service [12]. Since 2001, the RTA began inducting some volunteers aged 20 years or older in the military [13]. These males were not selected by the lottery system.

The inclusion criteria for this study included RTA conscripts aged at least 20 years and residing in military units in the central region (Bangkok and Lopburi Province), the northern region (Chiang Mai Province), the northeast region (Ubon Ratchathani Province) and the southern region (Songkhla Province) in the first week of May and November annually from 2009 to 2016.

### Data collection

In the first week after induction, in May and November annually, the conscripts body weight and height were measured to calculate the baseline BMI and to screen for high-risk groups for heat-related illness. A standardized case report form was used to obtain the required information from questionnaires, which was then sent to the Military Medicine Research Unit of Phramongkutklao College of Medicine in Bangkok. Data from all conscripts were collected using standardized questionnaires after the participants signed the consent form and covered information concerning demographic data, regular exercise (30 min/day and at least three days/week) and smoking. An ex-smoker was defined as being smoke-free for 12 months.

The participants’ body weight was measured in kilograms, and the height in centimeters. BMI was calculated as body weight in kilograms divided by height in meters squared (kg/m^2^). BMI scores were classified into four groups, including < 18.5 kg/m^2^ (underweight), 18.5 to 24.9 kg/m^2^ (normal range), 25.0 to 29.9 kg/m^2^ (overweight) and 30.0 kg/m^2^ (obese) [14].

### Statistical analysis

Data were coded and entered in STATA/MP for Windows, Version 12 (Stata Corp LP, TX, USA). Categorical data are presented as numbers and percentages, while continuous data are presented as the mean and standard deviation (SD). Prevalence was analyzed using descriptive statistics and reported as a percentage with a 95% confidence interval. The chi-square test was used to compare categorical data, while continuous data were compared using the *t*-test. Continuous data were grouped to analyze associated factors using the odds ratio (*OR*), while binary logistic regression analysis was used to determine the risk factors associated with obesity. The magnitude of association was presented as crude *OR* with a 95% confidence interval (CI). Multivariate analysis was performed using logistic regression analysis, and a *P* value less than 0.05 was considered statistically significant.

### Ethics consideration

The study was reviewed and approved by the Institutional Review Board of the RTA Medical Department. Written informed consent was obtained from the participants after they read the information sheet.

## Results

### Demographic characteristics

A total of 26,540 young Thai men were included in the study from 2009 to 2016. The age of the participants was (21.4 ± 1.2) years. Descriptive characteristics of the study participants by year are presented in Table 1. In all, 7058 (26.6%) participants were from the central region, 8364 (31.5%) came from the northern region, 5215 (19.6%) came from the Northeast region, and 5903 (22.2%) came from the southern region. The BMI of participants was (22.1 ± 3.5) kg/m^2^**.** One-fifth of the participants were students, while one in ten participants was unemployed; 20 % of all participants were agriculturists.

**Table 1:** Demographic characteristics of participants (*n* (%)). ^a^Mean ± Standard Deviation

Item	2009	2010	2011	2012	2013	2014	2015	2016
Age (years)^a^	21.3 ± 1.1	21.4 ± 1.2	21.5 ± 1.1	21.4 ± 1.1	21.5 ± 1.2	21.5 ± 1.2	21.4 ± 1.2	21.4 ± 1.2
Region								
Central	466 (22.8)	867 (30.8)	774 (23.3)	815 (27.7)	1006 (28.4)	836 (23.7)	936 (23.1)	1358 (31.7)
Northeast	278 (13.6)	439 (15.6)	702 (21.1)	819 (27.8)	675 (19)	811 (23)	714 (17.6)	777 (18.1)
North	828 (40.5)	1009 (35.8)	1141 (34.4)	1137 (38.6)	1141 (32.2)	896 (25.4)	1189 (29.3)	1023 (23.9)
South	473 (23.1)	500 (17.8)	703 (21.2)	172 (5.8)	723 (20.4)	990 (28)	1214 (30)	1128 (26.3)
BMI (kg/m^2^)	21.7 ± 3.1	21.8 ± 3.4	21.8 ± 3.1	22.0 ± 3.4	22.2 ± 3.4	22.3 ± 3.5	22.4 ± 3.8	22.5 ± 3.7
< 18.5	220 (10.8)	352 (12.5)	353 (10.6)	317 (10.8)	299 (8.4)	321 (9.1)	457 (11.3)	424 (9.9)
18.5–22.99	1279 (62.5)	1657 (58.9)	2037 (61.4)	1731 (58.8)	2086 (58.8)	2008 (56.8)	2139 (52.8)	2298 (53.6)
23–24.99	288 (14.1)	362 (12.9)	465 (14.0)	426 (14.5)	563 (15.9)	529 (15)	618 (15.2)	645 (15)
25–29.99	209 (10.2)	345 (12.3)	379 (11.4)	368 (12.5)	467 (13.2)	514 (14.5)	626 (15.4)	708 (16.5)
≥ 30	49 (2.4)	99 (3.5)	86 (2.6)	101 (3.4)	130 (3.7)	161 (4.6)	213 (5.3)	211 (4.9)
Occupation								
Unemployed	480 (23.6)	652 (23.2)	815 (25)	678 (23.3)	814 (23.4)	831 (23.8)	839 (22)	785 (19.6)
Student	392 (19.3)	560 (19.9)	719 (22)	650 (22.3)	751 (21.6)	763 (21.9)	794 (20.8)	904 (22.5)
Agriculture	417 (20.5)	506 (18)	610 (18.7)	530 (18.2)	720 (20.7)	708 (20.3)	632 (16.6)	720 (18)
Employee	195 (9.6)	225 (8)	275 (8.4)	225 (7.7)	277 (8.0)	258 (7.4)	308 (8.1)	318 (7.9)
Labor	133 (6.5)	190 (6.8)	194 (5.9)	220 (7.6)	259 (7.4)	234 (6.7)	260 (6.8)	304 (7.6)
Merchant	159 (7.8)	244 (8.7)	260 (8)	232 (8)	327 (9.4)	304 (8.7)	472 (12.4)	496 (12.4)
Others	259 (12.7)	431 (15.3)	389 (11.9)	378 (13)	331 (9.5)	387 (11.1)	505 (13.3)	484 (12.1)
Education								
Less than primary	18 (0.9)	25 (0.9)	29 (0.9)	16 (0.5)	30 (0.9)	21 (0.6)	22 (0.5)	24 (0.6)
Primary	437 (21.4)	579 (20.6)	557 (17)	486 (16.6)	571 (16.3)	571 (16.3)	760 (18.9)	726 (17)
Secondary	1451 (71.1)	1986 (70.8)	2469 (75.5)	2245 (76.8)	2615 (74.7)	2600 (74.0)	2978 (74.0)	3218 (75.5)
University or higher	135 (6.6)	214 (7.6)	215 (6.6)	178 (6.1)	284 (8.1)	321 (9.1)	264 (6.6)	293 (6.9)

### Prevalence of obesity among young Thai men in 2016

In 2016, the overall mean BMI among young Thai men was (22.5 ± 3.7) kg/m^2^. The prevalence of obesity was 4.8%. The prevalence of obesity among young Thai men by region was 4.9, 8.7, 3.3 and 2.6% in the central, north, northeast, and south, respectively. Regarding young Thai men, the prevalence of obesity was higher among those with a higher education level. Among young Thai men, the prevalence of obesity in those working in outdoor occupations (3.1% among those in agriculture and 2.5% in labor) was lower than that in men working in indoor occupations (5.1% among office workers, 5.4% among students, 6.3% among retail workers and 6.7% among the unemployed).

### Trends in the prevalence of obesity among young Thai men

From 2009 to 2016, the overall prevalence of obesity among young Thai men increased significantly by the year of the survey. Table 2 shows the trends in the prevalence of obesity for young Thai men by geographic region, education level and occupation. The prevalence of obesity increased from 2.2% in 2009 to 3.4% in 2010, 2.5% in 2011, 2.9% in 2012, 3.4% in 2013, 4.4% in 2014, 5.0% in 2015 and 4.8% in 2016 (*P* for trend < 0.0001).

**Table 2:** Prevalence of obesity among young Thai men 2009–2016 (%). ^a^Total prevalence of obesity calculated by applying direct standardized method with reference regions population in survey years

Item	2009	2010	2011	2012	2013	2014	2015	2016	*P for* trend
Region									
Central	2.8	3.8	3	2.9	3.6	4.5	6	4.9	0.0005
Northeast	1.1	2.5	1.4	1.5	2.1	3.1	2.5	3.3	0.0045
North	3.3	3.6	2.8	5.3	5.5	6.8	7.7	8.7	0.0000
South	1.3	3.8	3	2.9	2.4	3.7	3.9	2.6	0.2611
Occupation									
Unemployed	2.5	5.3	2.3	2.6	4.3	5.9	4	6.7	0.0050
Student	2.9	4.2	3.6	4.2	3.1	3.8	7	5.4	0.0000
Agriculture	1.9	2.1	1.3	2.7	2.3	3.5	3.5	3.1	0.0042
Employee	2.6	2.5	2.1	2.8	2.9	4.3	5.4	5.1	0.5800
Labor	1.5	3.1	3.3	2.7	5.1	4.3	2.9	2.5	0.0114
Merchant	3	5.8	4.1	3.2	7.7	7.7	10	6.3	0.0201
Others	2.7	4.4	3.6	6.1	5.1	5.9	6.1	7.2	0.0024
Education									
Less than primary	0	0	0	6.3	3.3	4.8	0	0	0.7239
Primary	2.3	2.1	2.5	2.3	2.3	3.2	3.9	4.7	0.0009
Secondary	2.6	3.5	2.6	3.4	3.9	4.9	5.4	4.8	0.0000
University or higher	0.7	7	2.8	5.6	4.6	4.4	7.2	7.5	0.0184
Total^a^	2.2	3.4	2.5	2.9	3.4	4.4	5.0	4.8	0.0000

**Fig. 1:**
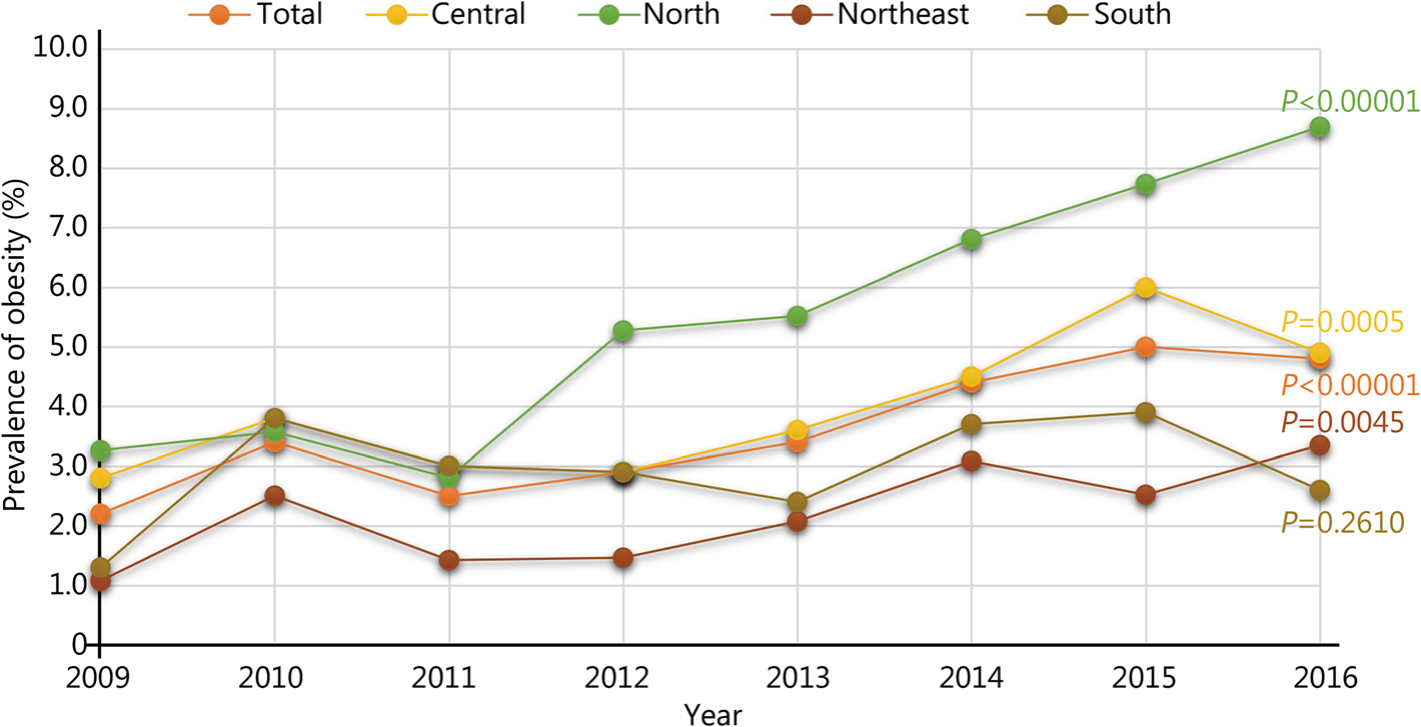
Prevalence of obesity by area of residence among young Thai men, from 2009 to 2016. *P for trend

**Fig. 2:**
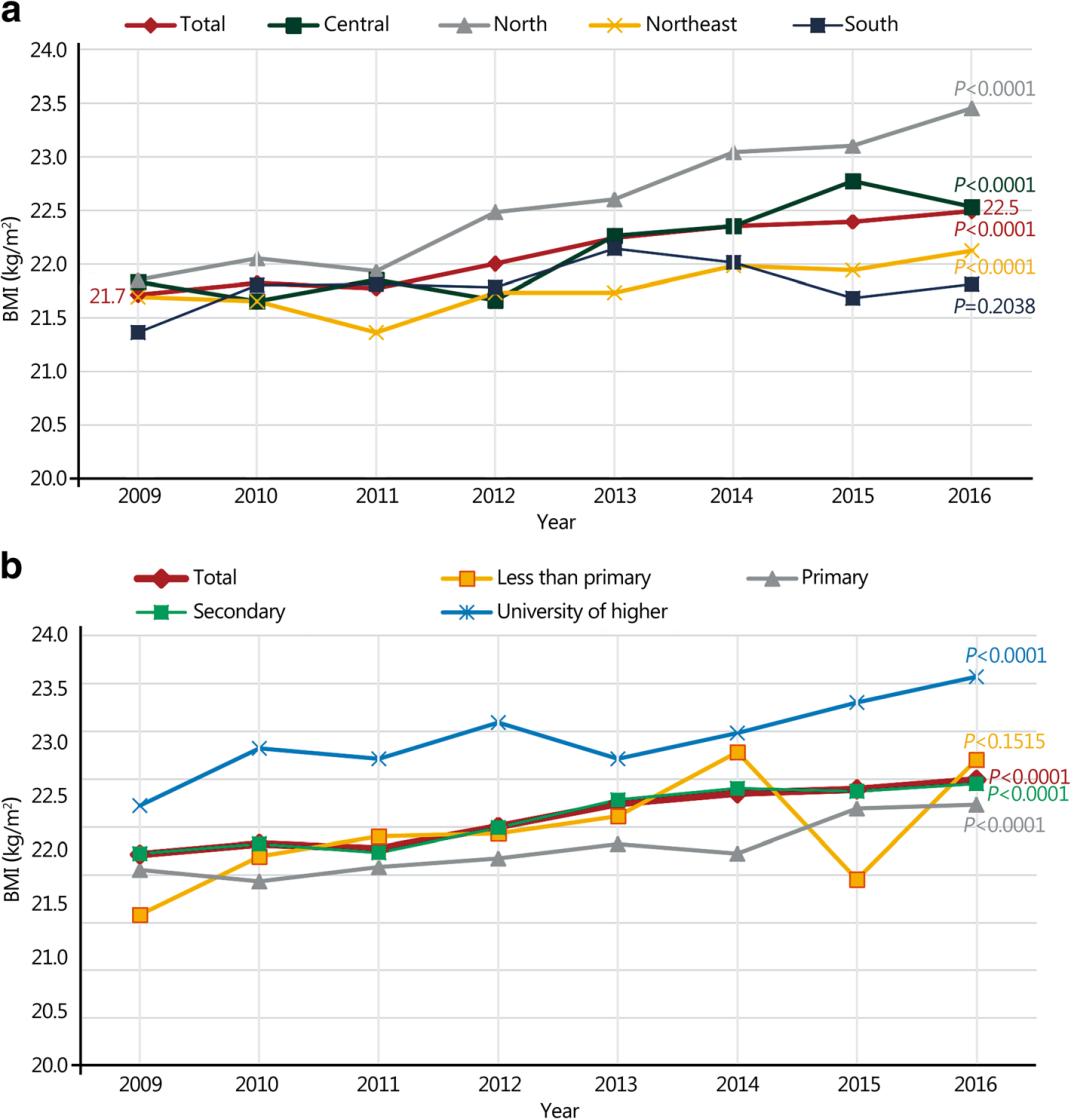
Average body mass index (BMI) by area of residence (**a**) and education level (**b**) among young Thai men, from 2009 to 2016. *P for trend

Figure 1 shows the trends in prevalence for young Thai men by geographic region. Obesity prevalence for all regions, except for the southern region, increased significantly from 2009 to 2016. Figure 2a and b show increasing trends in mean BMI by survey year according to geographic regions and education levels. Overall, the BMI trend for all subgroups increased to a certain extent. The BMI increased from (21.7 ± 3.1) kg/m^2^ in 2009 to (22.5 ± 3.7) kg/m^2^ in 2016, while the average increased BMI per 10 years was 1.0 kg/m^2^ (*P* < 0.0001). The average BMI increased across all educational levels. The rates of increase were highest among young men with less than primary education at 2.0 kg/m^2^ per decade.

### Associated factors of obesity among young Thai men

Univariate and multivariate logistic regression analyses were performed to determine factors associated with obesity. The final model was adjusted for survey year, age, geographic regions, education level, occupation, regular exercise and smoking. After adjusting for potential confounders, the factors associated with obesity included survey year, geographic regions, indoor occupation and regular exercise. Table 3 illustrates a significant increase in the annual prevalence of obesity. Young Thai men in the central, northern, and southern regions had an increased risk of obesity compared with those in the northeast region (adjusted odds ratios (AORs) 1.85, 2.65 and 1.37, respectively). Young Thai men with higher education, including secondary and university, were more likely to be obese compared with those with lower primary education. Young Thai men with indoor occupations and other occupations tended to be at high risk for obesity compared with those in outdoor occupation (AORs: 1.41; 95% CI: 1.18–1.67). In addition, we found that young Thai men without regular exercise had a higher risk of obesity than those who exercised regularly (AORs: 1.61; 95% CI: 1.39–1.86). Moreover, using subgroup analysis for factors regarding regular exercise, we found that young Thai men who regularly exercised (at least 30 min/day) ≥5 days/week were at a significantly lower risk of obesity (AORs: 0.689; 95% CI: 0.52–0.90) than those who exercised less frequently.

**Table 3:** Multivariate logistic regression analysis for factors associated with obesity among young Thai men 2009–2016 (n (%))

Variables	BMI < 30 kg/m^2^	BMI ≥ 30 kg/m^2^	AORs	95%CI	*P*-value
Year					
2009	1996 (97.6)	49 (2.4)	1		
2010	2716 (96.5)	99 (3.5)	1.34	0.91–1.98	0.135
2011	3234 (97.4)	86 (2.6)	1.06	0.72–1.56	0.762
2012	2842 (96.6)	101 (3.4)	1.28	0.88–1.88	0.200
2013	3415 (96.3)	130 (3.7)	1.49	1.03–2.16	0.035
2014	3372 (95.4)	161 (4.6)	1.75	1.21–2.53	0.003
2015	3840 (94.7)	213 (5.3)	2.22	1.55–3.18	< 0.001
2016	4075 (95.1)	211 (4.9)	2.18	1.52–3.12	< 0.001
Age (year)^a^	21.4 ± 1.2	21.5 ± 1.2	0.96	0.89–1.03	0.233
Region					
Northeast	5096 (97.7)	119 (2.3)	1		
Central	6768 (95.9)	290 (4.1)	1.85	1.45–2.35	< 0.001
North	7904 (94.5)	460 (5.5)	2.65	2.11–3.33	< 0.001
South	5722 (96.9)	181 (3.1)	1.37	1.06–1.78	0.017
Education					
≤ Primary	4727 (97.0)	145 (3.0)	1		
Secondary	18,767 (95.9)	795 (4.1)	1.36	1.11–1.66	0.003
University or higher	1804 (94.7)	100 (5.3)	1.92	1.36–2.70	< 0.001
Occupation					
^b^Outdoor	7755 (97.2)	220 (2.8)	1		
^c^Indoor	14,028 (95.7)	636 (4.3)	1.41	1.18–1.67	< 0.001
Others	2995 (94.7)	169 (5.3)	1.65	1.32–2.06	< 0.001
Exercise					
Yes	10,138 (96.9)	320 (3.1)	1		
No	13,733 (95.3)	679 (4.7)	1.61	1.39–1.86	< 0.001
Smoking					
Current	14,716 (96.1)	604 (3.9)	1		
Ex-smoker	4826 (95.8)	209 (4.2)	0.96	0.80–1.14	0.618
Never	4421 (96.2)	173 (3.8)	0.88	0.73–1.07	0.193

Adjusted for: year, age, region, education level, occupation, exercise and smoking.

^a^Mean ± Standard Deviation

^b^Outdoor occupation: agriculture and labor

^c^Indoor occupation: unemployed, student, employee, and merchant

## Discussion

This study represented the largest recent epidemiological study of obesity prevalence and associated factors for obesity among a large sample of young adult men in Thailand. These data provide important evidence of increasing trends in the prevalence of obesity among young Thai men from 2009 to 2016. Risk factors for obesity included survey year, geographic regions, indoor occupation and no regular exercise. The prevalence of obesity among young Thai men in 2016 was 4.8%; comparatively, a related report in China from 2010 to 2014 found an estimated prevalence of 5.4% [15]. A previous survey in Thailand in 2011 by Poston et al. [16], showed that the prevalence of obesity in young men was 2.1%. Additionally, the highest and lowest prevalence of obesity by region was 2.4 and 1.8% in the northern and southern regions, respectively, revealing a similar pattern as that found in the current study. The prevalence of obesity among young Thai men from 2009 to 2016 linearly increased with an average of 1.0 kg/m^2^ per decade and encompassed most geographic regions, education level, and occupations. The average BMI in young Thai men ranged from 21.7 kg/m^2^ to 22.5 kg/m^2^ from 2009 to 2016. This finding was similar to the related report in 2014 that reported that men had an age-standardized mean BMI ≥20 kg/m^2^ in every country [17]. Moreover, the average increase in BMI was higher than that of the global increase of 0.63 kg/m^2^ per decade [17]. Compared with other countries in Asia, the rise in BMI and prevalence of obesity in Thailand was consistent with the findings of other Asian countries, including China [15, 18], Korea [19], and Indonesia [20]. There was a dramatic increase in the prevalence of overweight and obesity among young Thai men from 12.6% in 2009 to 21.4% in 2016. Overweight and obesity have been confirmed to lead to health problems, including hypertension [4, 5], diabetes mellitus and especially atherosclerotic cardiovascular diseases (ASCVD). If overweight and obesity continue to progress, the number of patients with non-communicable diseases is more likely to increase. Thus, public health interventions should be provided early to young men to avoid increases in BMI.

The present study found that survey years were related to the prevalence of obesity. In the future, Thai society may be completely transformed to an industrial society. Everyone may consume fast food or high-energy food more than was consumed in the past, especially illiterate or low-income people who can access the mass-produced industrialized food, which is cheaper than traditional food, such as fruits and vegetables [21]. Then, high-energy food consumption occurs and fat will accumulate, causing obesity [22–24]. Consequently, we found that the prevalence of obesity was higher than that it was in 2009, and that geographic regions related to obesity prevalence. Young Thai men residing in the northeast are not as obese as those in other regions. This phenomenon can be explained by behavioral and local cultural factors. People in the northeastern regions may be more inclined to participate in their traditional festivities and traditional food, especially northeastern Thai food, which is rich in fruits and vegetables. Some people favor consuming a high fiber diet emphasizing vegetables and fruits, which may decrease body adiposity levels. Furthermore, traditional dancing and playing outdoor games are common in northeastern Thailand, while young Thai men in other regions may engage in fewer work-related physical activities and spend more time in sedentary activities, such as surfing the internet and playing games. Consequently, the young Thai men in the northeast region may engage in more physical activity than individuals in other regions. More physical activities can decrease body weight. One related study supported the finding that all physical activity burns calories, including spontaneous physical activity, which could be manipulated to burn calories, defend against weight gain and reduce excess adiposity [25]. Surprisingly, young adults with high education levels are more likely to gain more weight than those with lower education levels. Higher educated individuals tend to make more money from their occupation, so they can afford rich food more easily. In developed countries, richer people (with a better education) may eat healthier food and attend the gym or other venues for exercising. In developing countries such as Thailand, the richer people have access to more Western style foods, which are more energy dense.

Additionally, young adults with high education levels may study in the classroom, and this activity might involve inactive states leading to obesity [26, 27]. However, one study in Iran indicated that education level was inversely associated with obesity [28]. We found that young adults with indoor occupations tended to be obese more than those with outdoor occupations. Mostly, young people who worked indoors, including the unemployed, students, employees and merchants, had fewer physical activities and less active behavior compared with those working outdoors, including agriculturists and laborers. Moreover, agriculturalists may have little access to Western food, may have little money for buying food and have a more physically demanding job [26, 27]. Finally, we found that exercise served as a protective factor for obesity among young Thai men, especially those who exercised regularly at least five days weekly to decrease BMI significantly. Thus, young men who exercised regularly tended to present with obesity less often than those who did not exercising regularly [29, 30]. Consequently, if the process of obesity is terminated, the risks for non-communicable diseases will decrease.

Because the study employed a serial cross-sectional design, the results could show only the factors associated with obesity. The participants of this study comprised new conscripts who were recruited by the lottery system, and a few of the young men with a BMI greater than 35 kg/m^2^ were excluded from conscription. Therefore, this condition may have affected the results of this study by underestimating the prevalence of obesity. However, we could obviously observe significant increasing trends in the prevalence of obesity among young Thai men from 2009 to 2016. The strength of this study evaluating obesity among young men is the largest conducted in Southeast Asia. Thus, the findings of the study could be generalized and applied to other young male populations.

## Conclusions

Our data emphasized that obesity constituted a serious problem among young Thai men. We could apply these findings in military units to other groups at any age. Regular exercise should be provided to young adults and other age groups to reduce obesity; eventually, its associated complications, especially noncommunicable diseases could be erased.

## Abbreviations

AORs: Adjusted odds ratio
ASCVD: Atherosclerotic cardiovascular diseases
BMI: Body mass index
CI: Confident interval
HR: Hazard ratio
HRI: Heat related illness
ORs: Odds ratios
RTA: Royal Thai Army
SD: Standard deviation

## References

[1] Hruby A, Hu FB (2015). The epidemiology of obesity: a big picture. PharmacoEconomics..

[2] Ng M, Fleming T, Robinson M, Thomson B, Graetz N, Margono C (2014). Global, regional, and national prevalence of overweight and obesity in children and adults during 1980-2013: a systematic analysis for the global burden of disease study 2013. Lancet (London, England).

[3] Aekplakorn W, Inthawong R, Kessomboon P, Sangthong R, Chariyalertsak S, Putwatana P, et al. Prevalence and trends of obesity and association with socioeconomic status in Thai adults: national health examination surveys, 1991–2009. J Obes. 2014. 10.1155/2014/410259 PMC3976913 24757561

[4] DeMarco VG, Aroor AR, Sowers JR (2014). The pathophysiology of hypertension in patients with obesity. Nat Rev Endocrinol.

[5] Hall JE, do Carmo JM, da Silva AA, Wang Z, Hall ME (2015). Obesity-induced hypertension: interaction of neurohumoral and renal mechanisms. Circ Res.

[6] Danaei G, Singh GM, Paciorek CJ, Lin JK, Cowan MJ, Finucane MM, et al. The global cardiovascular risk transition: associations of four metabolic risk factors with macroeconomic variables in 1980 and 2008. Circulation. 2013:CIRCULATIONAHA. 113.001470. 10.1161/CIRCULATIONAHA.113.005449 24166421

[7] Collaboration PS (2009). Body-mass index and cause-specific mortality in 900 000 adults: collaborative analyses of 57 prospective studies. Lancet.

[8] Grundy SM (2004). Obesity, metabolic syndrome, and cardiovascular disease. J Clin Endocrinol Metab.

[9] Grundy SM, Hansen B, Smith SC, Cleeman JI, Kahn RA (2004). Clinical management of metabolic syndrome: report of the American Heart Association/National Heart, Lung, and Blood Institute/American Diabetes Association conference on scientific issues related to management. Circulation..

[10] Hirko KA, Kantor ED, Cohen SS, Blot WJ, Stampfer MJ, Signorello LB (2015). Body mass index in young adulthood, obesity trajectory, and premature mortality. Am J Epidemiol.

[11] Nutong R, Mungthin M, Hatthachote P, Ukritchon S, Imjaijit W, Tengtrakulcharoen P (2018). Personal risk factors associated with heat-related illness among new conscripts undergoing basic training in Thailand. PLoS One.

[12] Mason CJ, Kitsiripornchai S, Markowitz LE, Chanbancherd P, Supapongse T, Jugsudee A (1998). Nationwide surveillance of HIV-1 prevalence and subtype in young Thai men. Journal of acquired immune deficiency syndromes and human.

[13] Celentano DD, Nelson KE, Suprasert S, Eiumtrakul S, Tulvatana S, Kuntolbutra S (1996). Risk factors for HIV-1 seroconversion among young men in northern Thailand. Jama..

[14] Organization WH. Obesity: preventing and managing the global epidemic: World Health Organization; 2000. 11234459

[15] He Y, Pan A, Wang Y, Yang Y, Xu J, Zhang Y (2017). Prevalence of overweight and obesity in 15.8 million men aged 15–49 years in rural China from 2010 to 2014. Sci Rep.

[16] Poston WS, Haddock CK, Jahnke SA, Jitnarin N, Tuley BC, Kales SN (2011). The prevalence of overweight, obesity, and substandard fitness in a population-based firefighter cohort. J Occup Environ Med.

[17] Trends in adult body-mass index in 200 countries from 1975 to 2014: a pooled analysis of 1698 population-based measurement studies with 19.2 million participants. Lancet (London, England). 2016;387(10026):1377–96. 10.1016/S0140-6736(16)30054-X PMC7615134 27115820

[18] Sun J, Zhou W, Gu T, Zhu D, Bi Y (2018). A retrospective study on association between obesity and cardiovascular risk diseases with aging in Chinese adults. Sci Rep.

[19] Shin H-Y, Kang H-T (2017). Recent trends in the prevalence of underweight, overweight, and obesity in Korean adults: the Korean National Health and nutrition examination survey from 1998 to 2014. Journal of epidemiology.

[20] Rachmi C, Li M, Baur LA (2017). Overweight and obesity in Indonesia: prevalence and risk factors—a literature review. Public Health.

[21] Wallinga D (2009). Today's food system: how healthy is it?. Journal of hunger & environmental nutrition.

[22] Stelmach-Mardas M, Rodacki T, Dobrowolska-Iwanek J, Brzozowska A, Walkowiak J, Wojtanowska-Krosniak A (2016). Link between food energy density and body weight changes in obese adults. Nutrients..

[23] Garcia G, Sunil TS, Hinojosa P (2012). The fast food and obesity link: consumption patterns and severity of obesity. Obes Surg.

[24] Zhao Y, Wang L, Xue H, Wang H, Wang Y (2017). Fast food consumption and its associations with obesity and hypertension among children: results from the baseline data of the childhood obesity study in China mega-cities. BMC Public Health.

[25] Kotz CM, Perez-Leighton CE, Teske JA, Billington CJ (2017). Spontaneous physical activity defends against obesity. Curr Obes Rep.

[26] Mesas AE, Guallar-Castillon P, Leon-Munoz LM, Graciani A, Lopez-Garcia E, Gutierrez-Fisac JL (2012). Obesity-related eating behaviors are associated with low physical activity and poor diet quality in Spain. J Nutr.

[27] Pietiläinen KH, Kaprio J, Borg P, Plasqui G, Yki-Järvinen H, Kujala UM (2008). Physical inactivity and obesity: a vicious circle. Obesity..

[28] Hajian-Tilaki K, Heidari B (2009). Association of educational level with risk of obesity and abdominal obesity in Iranian adults. J Public Health.

[29] Ko I-G, Choi P-B (2013). Regular exercise modulates obesity factors and body composition in sturdy men. Journal of exercise rehabilitation.

[30] Swift DL, Johannsen NM, Lavie CJ, Earnest CP, Church TS (2014). The role of exercise and physical activity in weight loss and maintenance. Prog Cardiovasc Dis.

